# Gangrenous mastitis in dromedary camels in UAE caused by *Streptococcus agalactiae*

**DOI:** 10.1186/s12917-020-02382-8

**Published:** 2020-06-03

**Authors:** El Tigani Ahmed El Tigani-Asil, Ghada Elderdiri Abdelwahab, Jayamohanan Thikkal Veedu Payyan Veedu, Abdelmalik Ibrahim Khalafalla, Zayed Saud Abdullah Mohamed, Hassan Zackaria Ali Ishag, Asma Abdi Mohamed Shah, Mohamed Ali Abdulla Alhosani, Salama Suhail Mohammed Al Muhairi

**Affiliations:** Veterinary laboratories, Animal Wealth Sector, Abu Dhabi Agriculture and Food Safety Authority (ADAFSA), Abu Dhabi, United Arab Emirates

**Keywords:** Agalactiae, Gangrenous mastitis, Dromedary camels, *Streptococcus agalactiae*, 16S rRNA gene

## Abstract

**Background:**

Mastitis is a disease of economic concern that affects dairy industry worldwide. This study aimed to investigate and identify possible etiologies encountered in an episode of acute gangrenous mastitis in lactating she-camels in Al Dhafra region, Abu Dhabi Emirate, United Arab Emirates (UAE).

Beside the routine clinical examination, conventional bacteriological methods were used to isolate and identify possible aerobic/anaerobic bacterial or fungal pathogens from cultured milk samples collected from the mastitic she-camels. Moreover, quantitative real-time polymerase chain reaction (qPCR) was used for the detection of *Mycoplasma agalactiae* and *Mycoplasma bovis* strains, and the 16S rRNA gene was sequenced to confirm the isolation. The isolates were also tested for their susceptibility to antimicrobials.

**Results:**

Acute gangrenous mastitis is reported in the dromedary camel herd with about 80% morbidity rate among lactating she-camels exhibited acute, painful hard swelling of affected teat, quarter or entire udder. About 41.7% of the infected animals were stamped out for culling due to complete or partial amputation of udder quarters*. Streptococcus agalactiae* was the sole isolated organism (6 isolates). The antimicrobial susceptibility testing revealed that, the *Streptococcus agalactiae* isolates were sensitive to both penicillin and ampicillin. Comparison of the 16S rRNA gene sequencing results by BLASTN confirmed the presence of *Streptococcus agalactiae* with high confidence (100% identity). Phylogenetic analysis indicated clustering of one isolate (CM**A**UAE accession number; MN267805.1) with *Streptococcus agalactiae* that infects multi-hosts including humans, while strains (CM**B**UAE to CM**F**UAE with accession numbers; MN267806.1 to MN267810.1 respectively) clustered with *Streptococcus agalactiae* that infects humans. No *Mycoplasma spp* was detected by qPCR analysis.

**Conclusions:**

In the present study, the *Streptococcus agalactiae* was found to be the main cause of acute gangrenous mastitis in dromedary camels in UAE. More research should be done to investigate other possible causes of clinical or subclinical mastitis in dromedary camels in UAE.

## Background

Mastitis is an intramammary infection characterized by physical, chemical and bacteriological changes in the milk and pathological changes in the glandular tissue. It has a negative impact on human health and animal production.

Bacterial infections are considered the primary cause of mastitis in domestic animals. However, there is paucity of information about the etiological agents associated with camel mastitis. Few available literatures indicated the causative agents of mastitis in camel which include *Staphylococcus aureus*, *Streptococcus spp* [1–3], *Micrococcus spp, Streptococcus agalactiae* [1, 3], *Coagulase negative staphylococci* [1], *Staphylococcus epidermis*, *Pasteurella haemolytica* [4], *Escherichia coli* [1, 5] and *Corynebacterium spp* [2]. The forms of mastitis in camel can be clinical mastitis including (acute or chronic) or sub-clinical mastitis. Clinical mastitis is characterized by swelling, heat, pain in the mammary gland, and the milk is discoloured and clotted. In acute mastitis, the mammary secretions are watery, yellowish or blood-tinged and bacteria isolated have included *Klebsiella pneumoniae* and *E. coli* [2]. Chronic mastitis is characterized by fibrosis and keratinization of the udder tissue. In Saudi Arabia, Ramdan et al. (1987) described unilateral chronic mastitis caused by *Staph aureus* and *Mannheimia (Pasteurella) haemolytica* [6]. In subclinical mastitis, the animal does not exhibit typical mastitis symptoms mentioned above [7].

Mastitis is usually categorized into contagious and environmental. For the contagious type, udders serve as a reservoir for several pathogens including *Staphylococcus aureus*, *Streptococcus agalactiae*, *Mycoplasma Spp* and *Corynebacterium bovis*, which can spread primarily during milking [8]. The environmental type is caused by environmental pathogens living in soil, bedding, water, manure, calving pads. Examples of these bacteria include *E. coli*, *Klebsiella spp*, *Streptococcus dysagalactiae* and *Streptococcus uberis* [9]. These bacteria have been reported to cause mastitis in UAE [10] and other countries such as Iraq, Rwanda, Ethiopia and Kenya [11–14]. Some viruses were also reported to cause mastitis such as bovine herpesvirus II and IV [15].

There are several identified risk factors affecting the prevalence of mastitis including climate, season, bedding, housing system, herd population, husbandry practice and worker’s health status. Tick infestation could also predispose udder infection by creating a skin and teat lesions [16]. In this article, we investigated the possible pathogens that cause mastitis in she-camels in order to determine the proper therapy and to characterize it genetically.

## Results

### Clinical examination

**Fig. 1:**
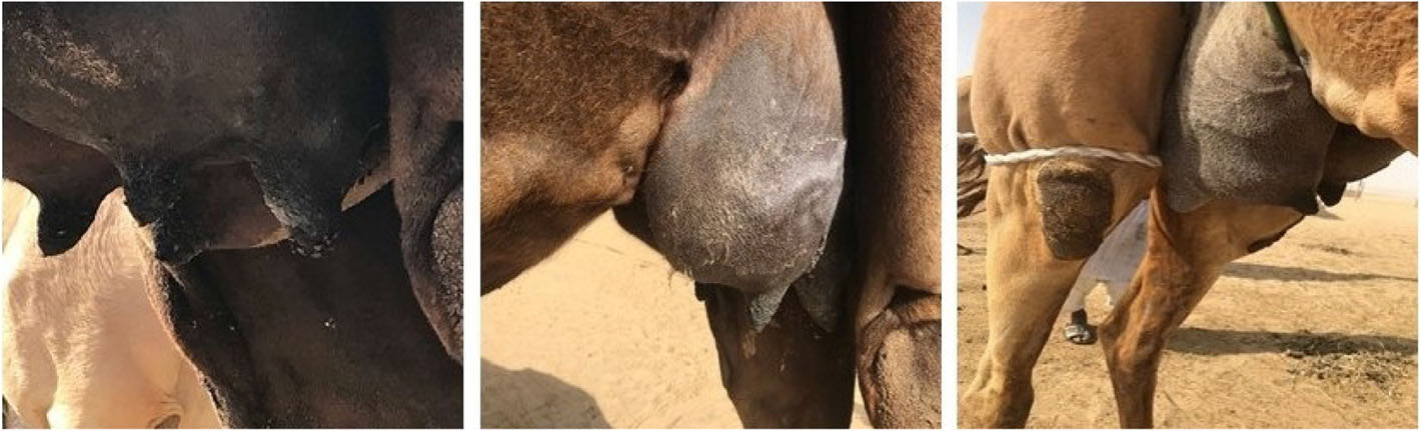
Acute swelling of udder quarters and formation of scabs

**Fig. 2:**
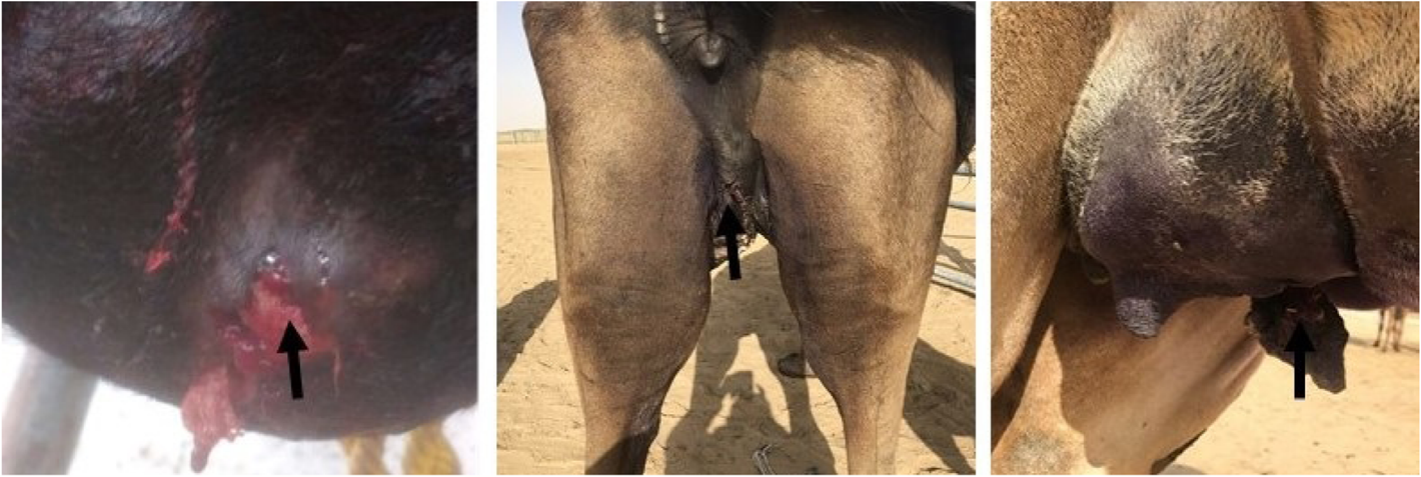
Acute swelling of udder with gangrenous inflammation and amputation of teats, quarters or entire udder (arrows)

Clinical examination of the infected camel herd showed acute and rapidly disseminated episode of gangrenous mastitis in 80% of lactating she-camels during the period from January to February 2018. Acute clinical signs of painful swelling, redness, hardening of the udder and failure to produce milk were observed (Fig. 1). Physical and chemical changes in the properties of milk include bloody milk, watery consistency and flakes formation also observed. Acute gangrenous mastitis was observed in 5/12 (41.7%) animals out of the total infected population. Three she-camels (60%) partially lost some udder quarters, while two of the animals (40%) lost their entire udder. Mastitis was rapidly developed into severe necrosis, suppuration, gangrenous inflammation with blacking, coldness, corrugation of udder skin and putrefaction, which were terminated with sloughing or amputation of affected quarters and sometimes the entire udder (Fig. 2). The diseased animals were responded to systemic and intramammary penicillin treatment.

### Conventional bacteriological methods

**Fig. 3:**
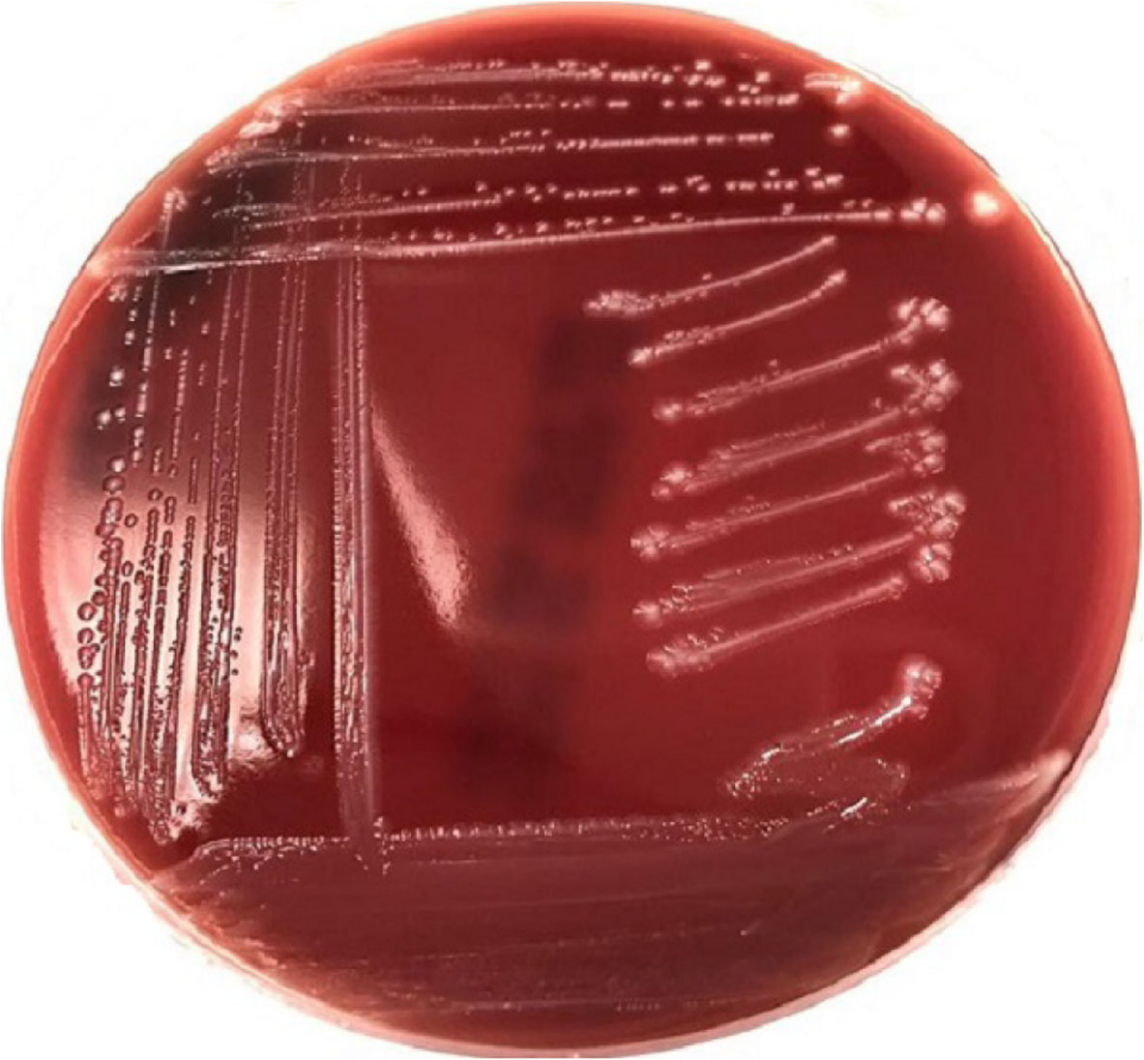
Colony morphology of *Streptococcus agalactiae*, blood agar 24 h at 37 °C

**Fig. 4:**
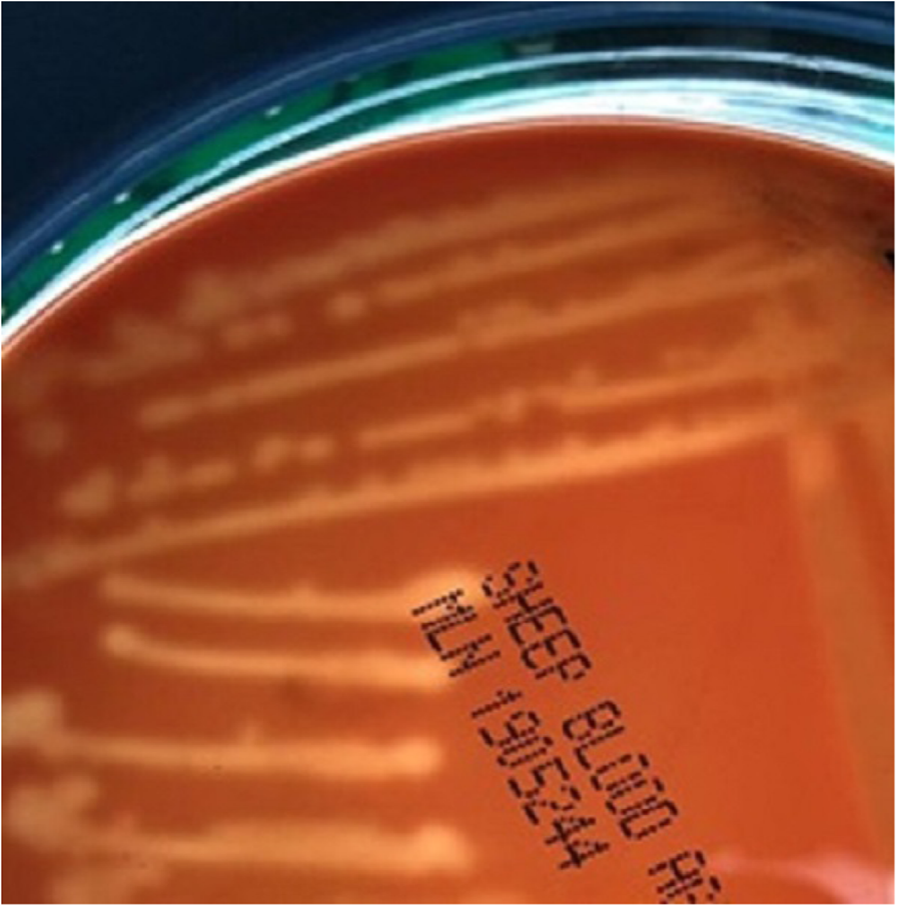
*β*-hemolytic colonies of *Streptococcus agalactiae*, blood agar 24 h at 37 °C

**Fig. 5:**
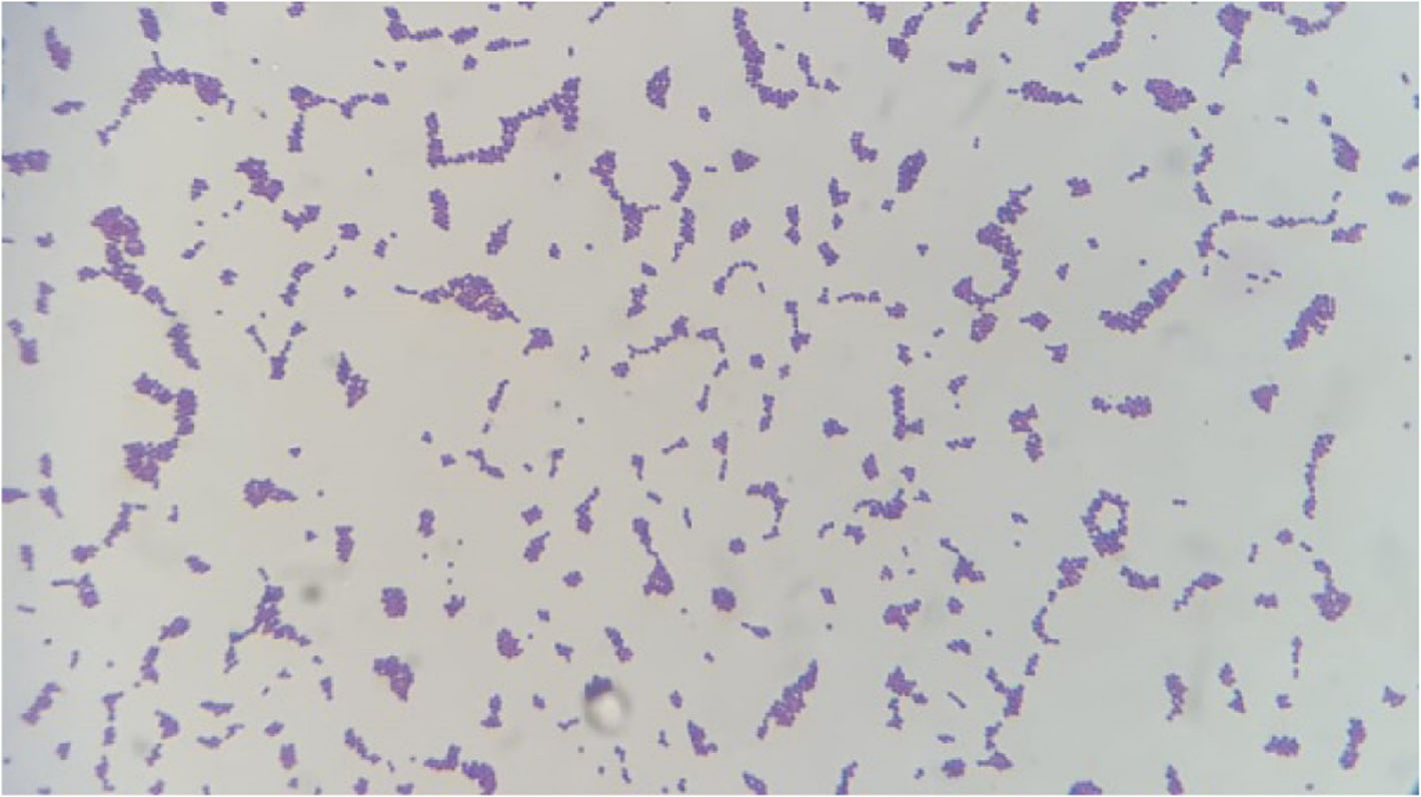
Gram stain film showing single and short chains of *Streptococcus spp*

Aerobic and anaerobic incubation of inoculated milk samples on blood agar revealed pure growth of small translucent (Fig. 3) beta-hemolytic colonies with approximately 1 mm in diameter (Fig. 4). The isolates are phenotypically gram-positive cocci in single and short chains (Fig. 5) and identified as *Streptococcus agalactiae* (6 isolates) by the Api 20 strep and Vitek II identification systems (BioMérieux). Catalase test was performed and found to be negative. Additionally, no fungus or yeast were isolated.

### Screening for *Mycoplasma spp* by qPCR

In order to exclude the other common possible pathogenic bacteria of mastitis, molecular detection of *Mycoplasma agalactiae* and *Mycoplasma bovis* was performed, and the results were negative (data not shown).

### Screening for viruses

No virus was isolated in cell culture from five skin scraping homogenates. The PCR was also negative for Ortho- and Parapoxviruses.

### Antimicrobial sensitivity test

The disc diffusion method on Muller-Hinton agar was performed to examine sensitivity reactions of the isolates against penicillin (10 U) and ampicillin (10 μg). The results indicated that, the six isolates of *Streptococcus agalactiae* are sensitive to both penicillin and ampicillin under the above-mentioned concentrations.

### 16S rRNA gene sequencing, BLAST and phylogenetic analysis

**Fig. 6:**
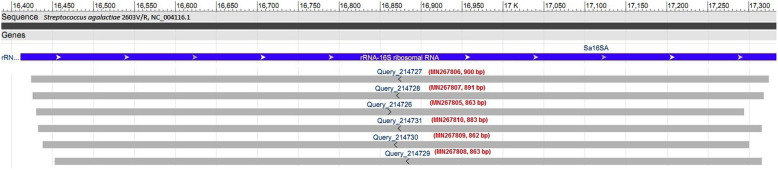
Mapping of the isolates (accession numbers: MN267805.1 to MN267810.1) to the reference genome (*Streptococcus agalactiae* 2603 V/R, NC_004116.1). Mapping and visualization performed with NCBI-BLASTN services

The 16S rRNA gene was sequenced to confirm the bacterial isolation. The partial sequences of the 16S rRNA gene (862–900 bp) of the six isolates were compared to the known nucleotide sequences in the GenBank database using BLASTN network services. Sequence alignments identified the genus and species as *Streptococcus agalactiae* with very high scores of identities (100%) and significance (e-value of 0.0) (see Additional files 1, 2, 3, 4, 5 and 6). These sequences of the six isolates were deposited in the GenBank under the names CM**A**UAE, CM**B**UAE, CM**C**UAE, CM**D**UAE, CM**E**UAE and CM**F**UAE, and the accession numbers shown in (Table 1). The sequence of six isolates of *Streptococcus agalactiae* were also mapped against the reference genome of *Streptococcus agalactiae* (accession number AE008948.1) using the NCBI network services. Mapping of the isolates to the 16S rRNA region of the reference genome has been shown in Fig. 6, while the span of the coverage regions was shown in Table 2.

**Table 1:** GenBank accession numbers from the 16S rRNA gene partial sequence of *Streptococcus agalactiae* isolated from she-camels collected milk with mastitis

GenBank submission	Isolate sequence (strain)	Accession number
SUB6137525	Seq1 (CMAUAE)	MN267805.1
SUB6137525	Seq2 (CMBUAE)	MN267806.1
SUB6137525	Seq3 (CMCUAE)	MN267807.1
SUB6137525	Seq4 (CMDUAE)	MN267808.1
SUB6137525	Seq5 (CMEUAE)	MN267809.1
SUB6137525	Seq6 (CMFUAE)	MN267810.1

**Table 2:** Mapping of the isolates (accession numbers: MN267805.1 to MN267810.1) against reference genome (*Streptococcus agalactiae* 2603 V/R, NC_004116.1). Span of mapped isolates including identity information were shown

Accession number/locus tag	Span		Length (bp)	Identity
	Start	End		
Sa16SA (16S rRNA gene of Ref. genome, complete)	16,411	17,917	1507	
MN267806 (16S rRNA gene of isolate1, partial)	16,425	17,324	900	100%
MN267807 (16S rRNA gene of isolate 2, partial)	16,427	17,317	891	100%
MN267805 (16S rRNA gene of isolate 3, partial)	16,431	17,293	863	100%
MN267810 (16S rRNA gene of isolate 4, partial)	16,433	17,315	883	100%
MN267809 (16S rRNA gene of isolate 5, partial)	16,439	17,300	862	100%
MN267808 (16S rRNA gene of isolate 6, partial)	16,453	17,315	863	100%

**Fig. 7:**
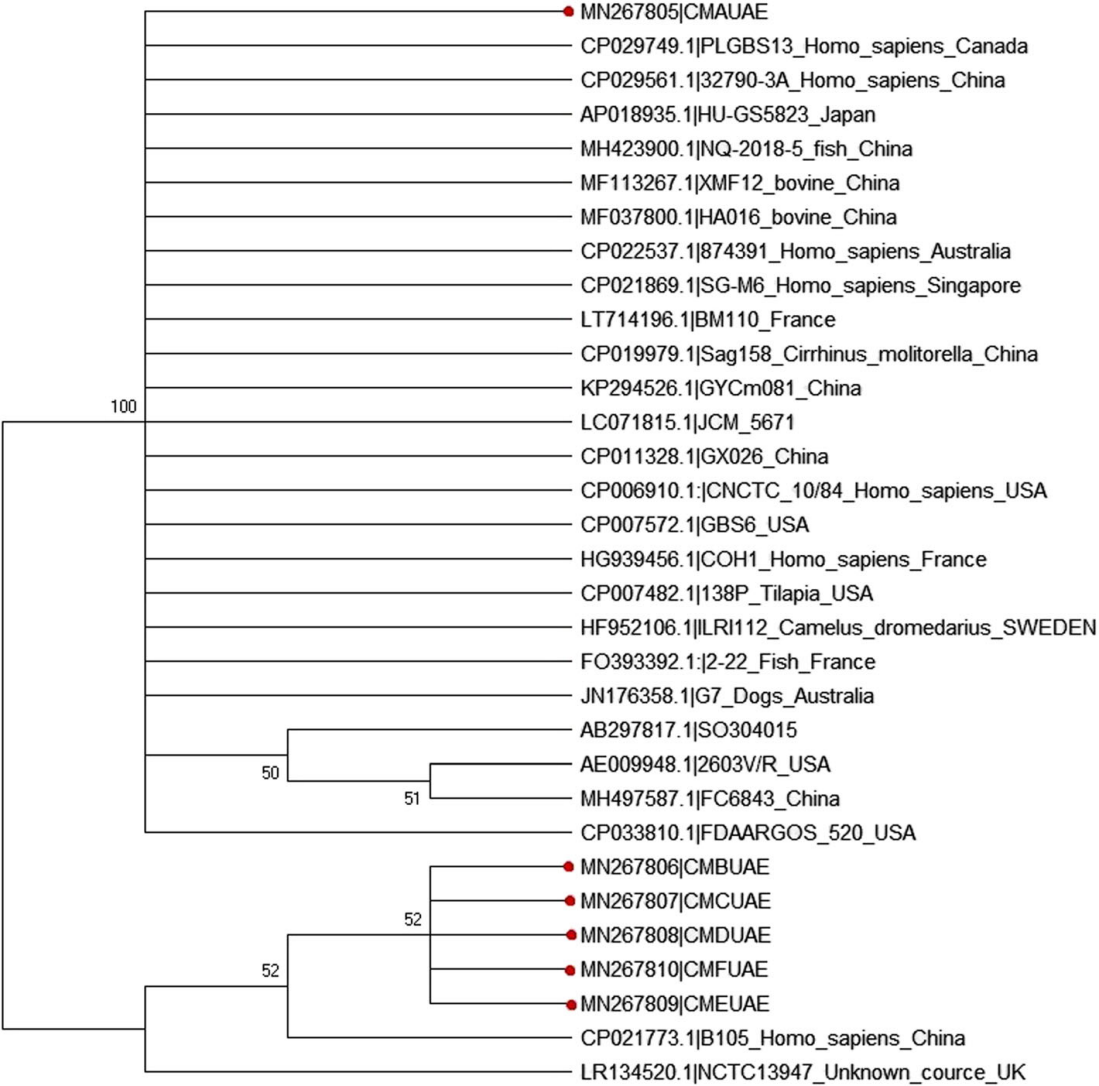
Phylogenetic analysis of 16S rRNA gene partial sequences. Phylogenetic analysis of partial sequence (862 bp) of 16S rRNA gene sequence was constructed with Neighbor-Joining method [17]. The optimal tree with sum of branch length = 1.11754156 is shown. The evolutionary distances were computed using the Kimura 2-parameter method [18]. Analysis involved 32 nucleotide sequences. Evolutionary analyses were conducted in MEGA X with Bootstrap test at 1000 replicates. Sequences from the present study are colored. Sequences from GenBank were given the accession number followed by the name of the circulated strain, species infected and then the country name

Phylogenetic analysis revealed that CM**A**UAE isolate is grouped with strains circulating in different regions in the world, including Canada, China, USA, Japan, Australia, Singapore, France, and Sweden while the rest of UAE isolates (CM**B**UAE to CM**F**UAE) clustered with B105 strain circulating in China and they were separated from other isolates with high bootstrap (100) value (Fig. 7).

## Discussion

Mastitis is an economically important disease of domestic animals, caused by several etiological agents predominantly bacterial species. In the present investigation, *Streptococcus agalactiae* is identified as a main cause of an outbreak of acute gangrenous mastitis in dromedary camels in UAE. These results are in concordance with the finding of other studies that likewise identified *Streptococcus spp* beside other bacterial pathogens as causes of sporadic clinical and subclinical mastitis of dromedary camels in UAE [10], in eastern Sudan [19] and in Kenya [14]. However, Al-Tofaily [11], reported *Mannheimia hemolytica* as a major cause of gangrenous mastitis in the she-camels in UAE. It is concluded that like in other animal species gangrenous mastitis in dromedary camels could be brought about by more than one pathogen.

The correlation between mastitis and *Streptococcus agalactiae* is well-documented. It has been reported that *Streptococcus agalactiae* was isolated from a healthy or sub-clinically infected camels as well in the pre-milking cows heifers [5, 12, 14, 20, 21], isolated from lactating camels (*Camelus dromedarius*) with mastitis [22], and isolated from a female llama (*Lama glama*) with chronic suppurative subcutaneous infection [23].

The penicillin and ampicillin antibiotics are drugs of choice as *Streptococcus agalactiae* isolates demonstrated to be sensitive to these antibiotics which is in accordance with the results showed previously by Hawari and Hassawi [24]. It is also important to pay a good attention to hygiene and proper management in order to minimize the occurrence of mastitis in the herd.

To study the evolution and zoonotic potential of the isolated *Streptococcus agalactiae*, we constructed a phylogenetic tree with (862–906 bp) of 16S rRNA gene sequences along with other selected 16S rRNA gene sequences derived from the GenBank. One of the isolates reported here; CM**A**UAE clustered with multi-species strains of *Streptococcus agalactiae* shown to infect humans, bovine, camels as well as dogs. Such multi-species infection of the pathogen was explained by the variations in the capsular structure of the bacteria [25]. The rest five UAE *Streptococcus agalactiae* isolates (CM**B**UAE to CM**F**UAE) together with the strain which infects human originating from China (CP021773.1|B105_Homo_sapiens_China) are gathered independently from other strains*.* This may demonstrate the possibility of the zoonotic transmission of the organism from camels and the possibility of occurrence of a new genotype of the pathogen adapted from human and circulated in camels in UAE. However, this should be further confirmed using complete 16S rRNA gene sequences. To the best of our knowledge, this is the first report in UAE on phylogenetic analysis based on the 16S rRNA gene sequence of *Streptococcus agalactiae* isolated from a she-camel with gangrenous mastitis.

Some limitations in this study include: the number of samples processed was inadequate to reveal the prevalence of camel mastitis in UAE and it is contagious nature, inability to collect samples for histopathology (live animal) and a limited number of herds investigated. In general, mastitis is considered one of the most significant constraints in the dairy sector in view of the enormous economic losses in the milk, culling of infected animals exhibiting complete amputation of udders and may have zoonotic impacts. More epidemiological investigations are required in order to determine the pathogens causing the disease, understand the possible risk factors and to determine the economic and public health impact of the disease.

## Conclusions

*Streptococcus agalactiae* could be considered as one of the causative agents of gangrenous mastitis in dromedary camels in UAE. Further studies are needed to determine the prevalence of the disease in UAE, characterize the causative bacteria and determine host-pathogen factors responsible for the development of the infection to gangrenous mastitis.

## Methods

### Study animals and clinical examination

An episode of acute mastitis form was noticed in one breeding camel herd at Eastern area of AL Dhafra region, emirate of Abu Dhabi, UAE in January–February 2018 during the routine follow-up by the veterinarians. The total number of camels in this herd was estimated to 100 animals including lactating she-camels managed under traditional open farm system for breeding purposes. The visual and manual examination of the infected animals was performed, and the pathological observations were recorded.

### Sample collection

Of the total infected she-camels (*n* = 12) aged 5–8 years, 7 samples of 10 mL milk were aseptically collected in sterile containers, and aliquoted for bacterial and fungal investigation, as well as molecular investigation. For the viral investigation, beside the milk samples, skin scrapings were collected from infected udders in sterile vials from five lactating camels. All the samples were kept at 4 °C during collection, transportation and preparation.

### Conventional bacteriological methods

Milk samples were mixed gently and a 10 μl loop full of milk was streaked onto Blood agar, MacConkey and SDA agars medium according to the standard microbiology culture procedures [26, 27]. Inoculated plates were incubated aerobically and anaerobically for possible isolation of any bacterial or fungal pathogens as described by Markey et al. [26]. Gram stain and catalase tests were performed to distinguish and classify the isolated bacteria. For quality control assay of catalase test, positive control of *Staphylococcus aureus* ATCC 25923 and negative control of *Enterococcus faecalis* ATCC 29212 were incorporated. Biochemical identification was done by using Api 20 strept and Vitek II identification systems (bioMérieux) following the manufacturer guidelines.

### Antimicrobial sensitivity test

Antimicrobial sensitivity test was done using the disc diffusion method on Muller-Hinton agar as described by CLSI 2018 [28] to examine sensitivity reactions of the isolates against penicillin (10 U) and ampicillin (10 μg). Quality control of the test was done by using *Streptococcus pneumoniae* ATCC 49619 as a positive control according to the same reference.

### Screening for *Mycoplasma Spp*

To further investigate the possibility of contribution of *Mycoplasma bovis*, and *Mycoplasma agalactiae* to the development of mastitis, the samples were outsourced to the Central Veterinary Research Laboratory (CVRL), Dubai, UAE for qPCR analysis.

### Screening for viruses

For viral isolation, skin scrapings were homogenized in the presence of sterile phosphate buffered saline and antibiotics and inoculated onto bovine fetal kidney (BFK) cells. DNA was extracted from the skin scrapings and subjected to PCR targeting the Parapoxvirus (PPV) major envelope protein (B2L) gene and the acidophilic type inclusion body protein (ATIP) gene of the Orthopoxvirus (OPXV) as previously described [29].

### 16S rRNA gene sequencing, BLAST and phylogenetic analysis

Six positive isolates identified as *Streptococcus agalactiae* by conventional bacteriological methods, were tested for species identification with 16S rRNA gene sequencing method. Universal 16S rRNA gene primers of 27F (5′-AGAGTTTGATCCTGGCTCAG-3′) and 1392R (5′-GGTTACCTTGTTACGACTT-3′) were used for PCR amplification and sequencing. Sanger sequencing was performed with BigDye® Terminator v3.1 Sequencing Standard Kit (Applied Biosystem) according to manufacturer instructions in SeqStudio Genetic Analyzer sequencer (Applied Biosystem). Each sample was sequenced in replicates with forward and reverse primers to increase bases confidence.

The raw sequence reads were trimmed, assembled into contig and the consensus sequence was compared with nucleotide sequence in the GenBank database by BLASTN, National Center for Biotechnology information (NCBI) to establish the closest known relative species. The phylogenetic analysis was constructed using MEGA X with Neighbor-Joining method [17] and evolutionary distances were computed using the Kimura 2-parameter model [18] with 1000 bootstrap replications.

## Supplementary information

**Additional file 1: **Top Ten BLAST alignment of isolate 1 (strain: CM**A**UAE, Acc. No. MN267805.1).

**Additional file 2: **Top Ten BLAST alignment of isolate 2 (strain: CM**B**UAE, Acc. No. MN267806.1).

**Additional file 3: **Top Ten BLAST alignment of isolate 3 (strain: CM**C**UAE, Acc. No. MN267807.1).

**Additional file 4: **Top Ten BLAST alignment of isolate 4 (strain: CM**D**UAE, Acc. No. MN267808.1).

**Additional file 5: **Top Ten BLAST alignment of isolate 5 (strain: CM**E**UAE, Acc. No. MN267809.1).

**Additional file 6: **Top Ten BLAST alignment of isolate 6 (strain: CM**F**UAE, Acc. No. MN267810.1).

## Data Availability

The datasets generated and/or analyzed during the current study are available in the GenBank repository, MN267805.1, MN267806.1, MN267807.1, MN267808.1, MN267809.1 and MN267810.1″.

## Abbreviations

UAE: United Arab Emirates
ADAFSA: Abu Dhabi Agriculture and Food Safety Authority
qPCR: Quantitative real-time polymerase chain reaction
rRNA: Ribosomal RNA
BLASTN: Basic Local Alignment Search Tool for nucleotide
24 h: 24 h
CMAUAE: Camel Milk number A, United Arab Emirates
CMBUAE: Camel Milk number B, United Arab Emirates
CMCUAE: Camel Milk number C, United Arab Emirates
CMDUAE: Camel Milk number D, United Arab Emirates
CMEUAE: Camel Milk number E, United Arab Emirates
CMFUAE: Camel Milk number F, United Arab Emirates
CVRL: Central Veterinary Research Laboratory
BFK: Bovine fetal kidney
PPV: Parapoxvirus
SDA: Sabouraud Dextrose Agar
CLSI: Clinical and Laboratory Standards Institute
