# Supplementary figures and images for: Gangrenous mastitis in dromedary camels in UAE caused by Streptococcus agalactiae
Source: BMC Vet Res. 2020 Jun 3;16:174. doi: 10.1186/s12917-020-02382-8 (PMC7271422; doi:10.1186/s12917-020-02382-8)

**
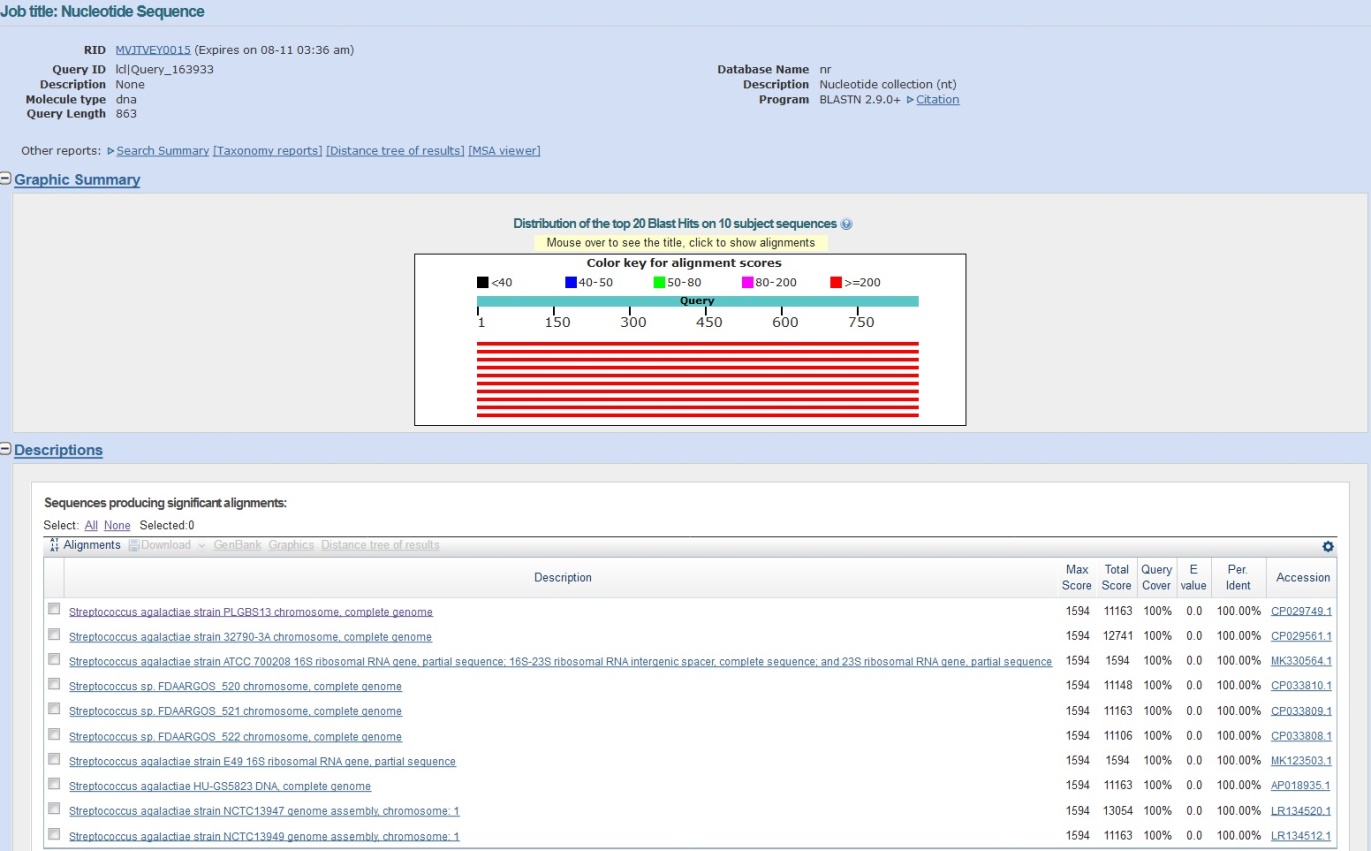
**

Additional file 1: Top Ten BLAST alignment of isolate 1 (strain: CM**A**UAE, Acc. No. MN267805.1)

Supplement: Supplementary file 1 — Additional file 1: Top Ten BLAST alignment of isolate 1 (strain: CMAUAE, Acc. No. MN267805.1). [file 12917_2020_2382_MOESM1_ESM.docx]

Additional file 2: Top Ten BLAST alignment of isolate 2 (strain: CM**B**UAE, Acc. No. MN267806.1)


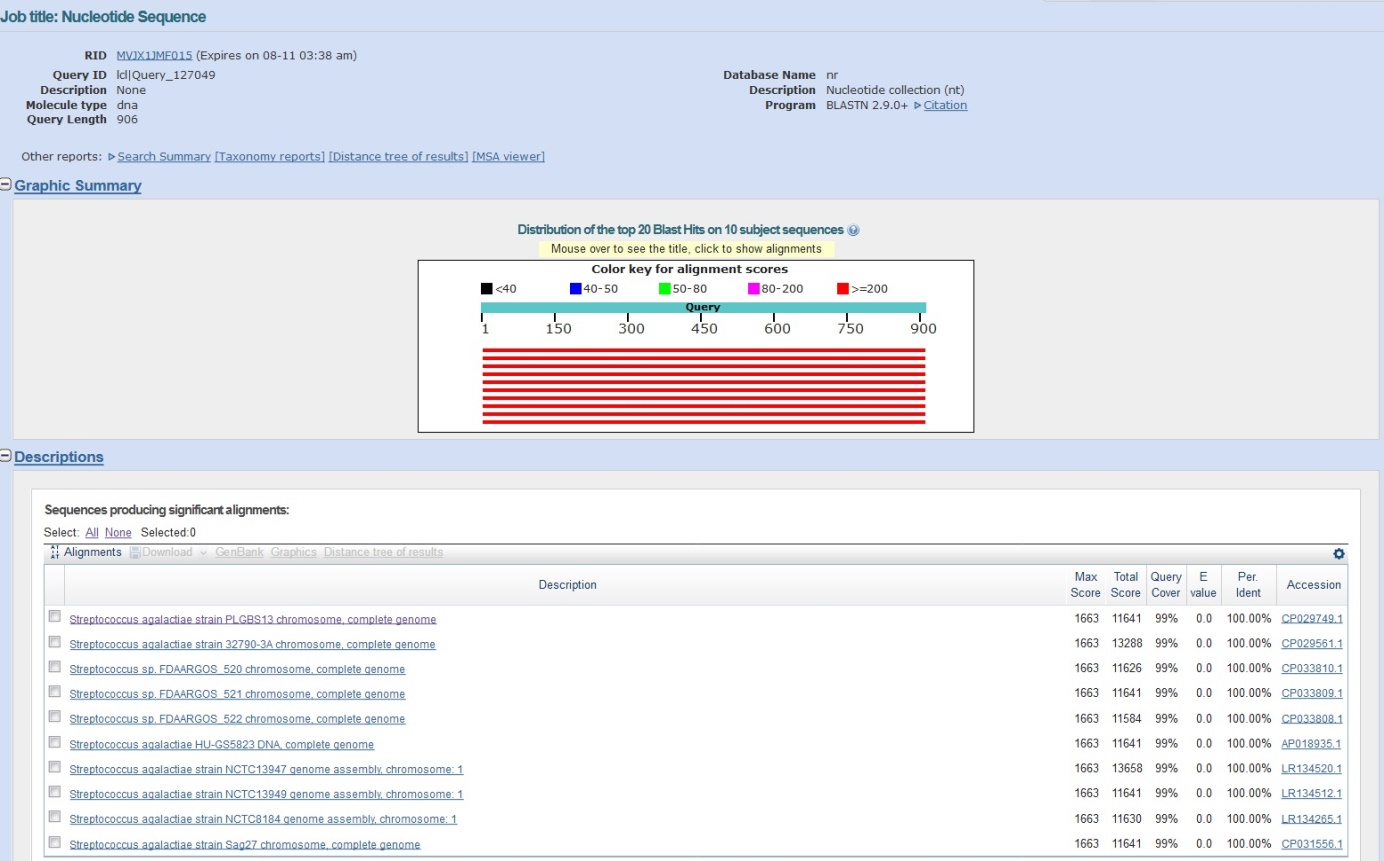

Supplement: Supplementary file 2 — Additional file 2: Top Ten BLAST alignment of isolate 2 (strain: CMBUAE, Acc. No. MN267806.1). [file 12917_2020_2382_MOESM2_ESM.docx]

Additional file 3: Top Ten BLAST alignment of isolate 3 (strain: CM**C**UAE, Acc. No. MN267807.1)
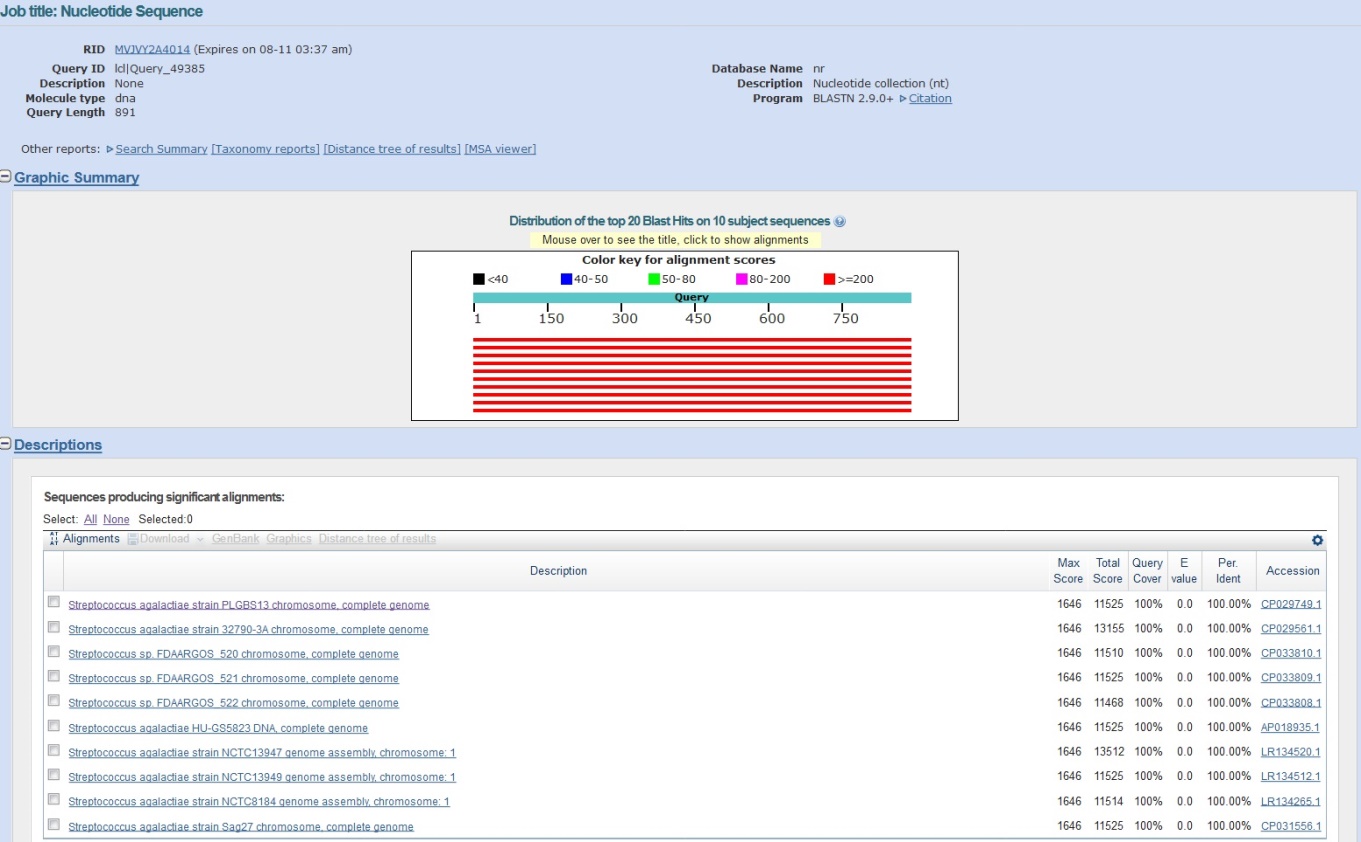

Supplement: Supplementary file 3 — Additional file 3: Top Ten BLAST alignment of isolate 3 (strain: CMCUAE, Acc. No. MN267807.1). [file 12917_2020_2382_MOESM3_ESM.docx]

Additional file 4: Top Ten BLAST alignment of isolate 4 (strain: CM**D**UAE, Acc. No. MN267808.1)
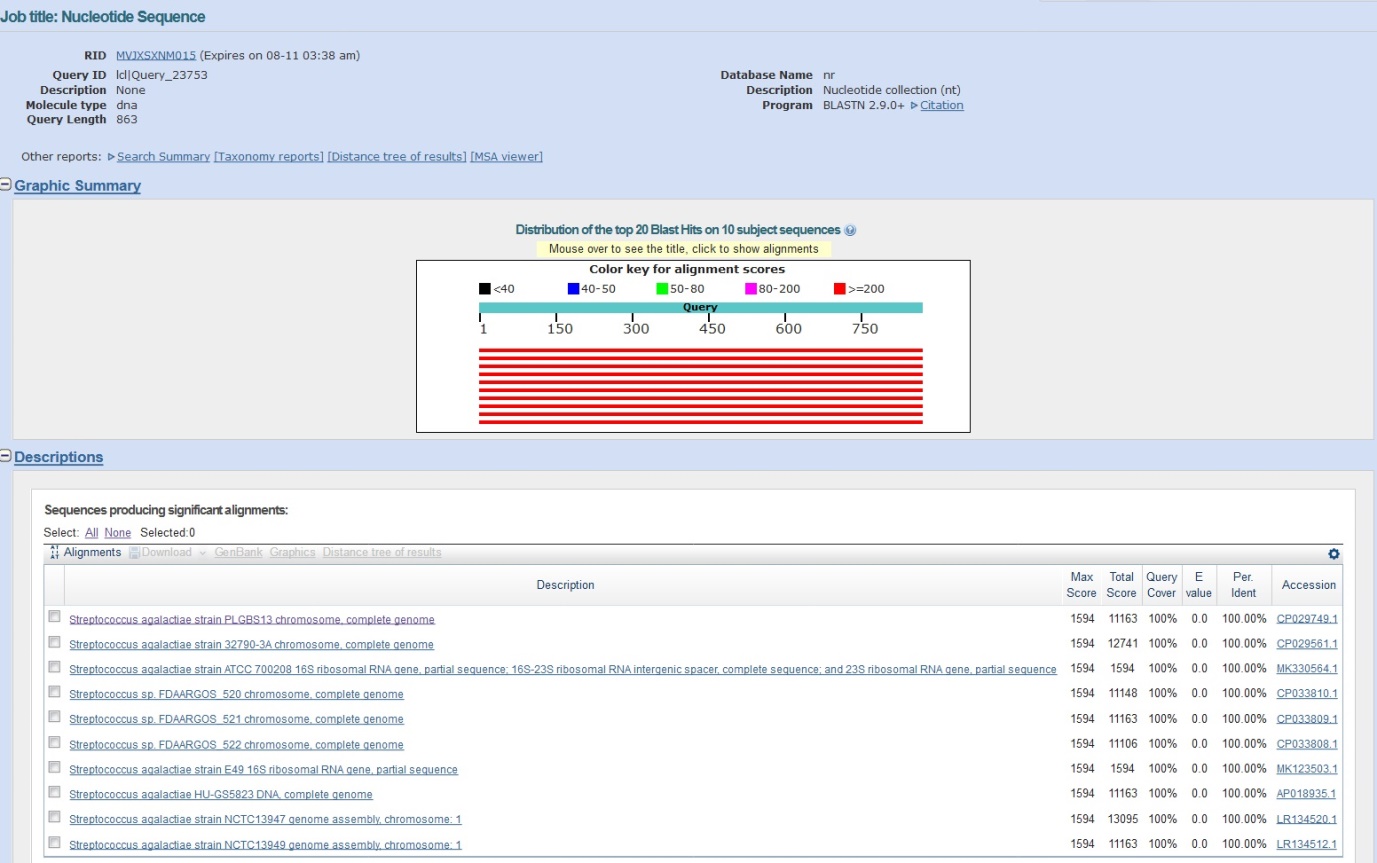

Supplement: Supplementary file 4 — Additional file 4: Top Ten BLAST alignment of isolate 4 (strain: CMDUAE, Acc. No. MN267808.1). [file 12917_2020_2382_MOESM4_ESM.docx]

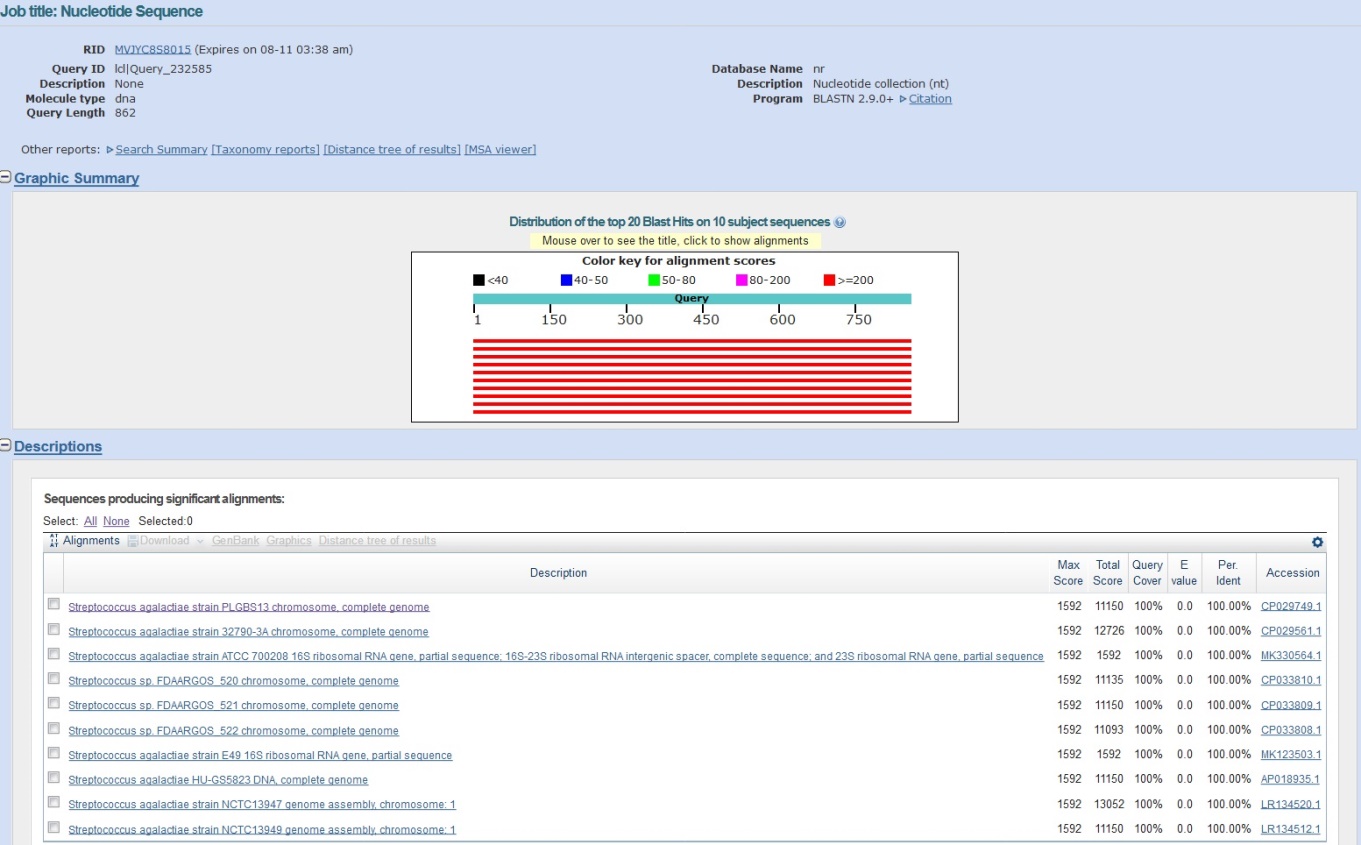
Additional file 5: Top Ten BLAST alignment of isolate 5 (strain: CM**E**UAE, Acc. No. MN267809.1)

Supplement: Supplementary file 5 — Additional file 5: Top Ten BLAST alignment of isolate 5 (strain: CMEUAE, Acc. No. MN267809.1). [file 12917_2020_2382_MOESM5_ESM.docx]

Additional file 6: Top Ten BLAST alignment of isolate 6 (strain: CM**F**UAE, Acc. No. MN267810.1)


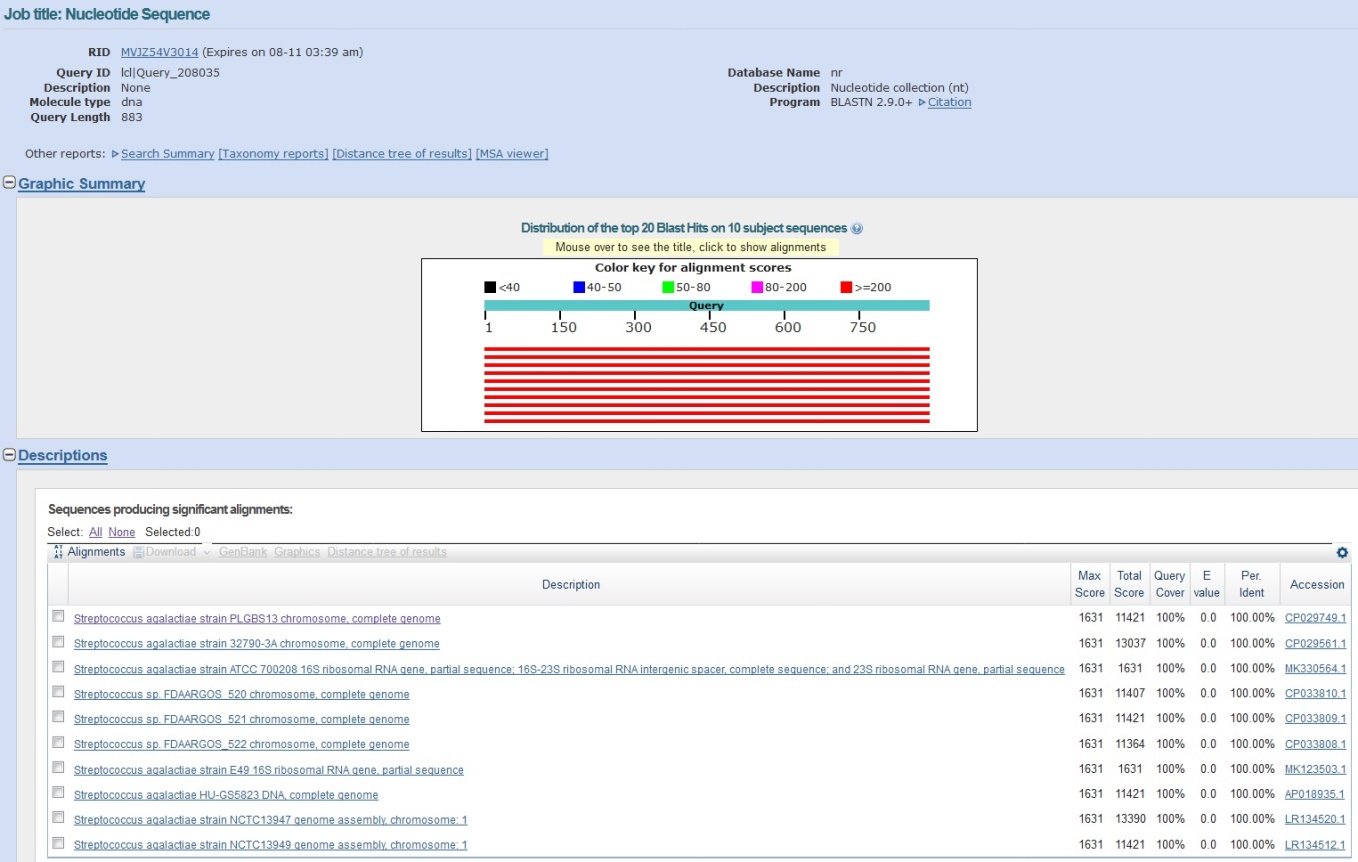

Supplement: Supplementary file 6 — Additional file 6: Top Ten BLAST alignment of isolate 6 (strain: CMFUAE, Acc. No. MN267810.1). [file 12917_2020_2382_MOESM6_ESM.docx]
